# Deep learning in CT image segmentation of cervical cancer: a systematic review and meta-analysis

**DOI:** 10.1186/s13014-022-02148-6

**Published:** 2022-11-07

**Authors:** Chongze Yang, Lan-hui Qin, Yu-en Xie, Jin-yuan Liao

**Affiliations:** 1 Department of Radiology, First Affiliated Hospital of Guangxi Medical University, Nanning, 530021 Guangxi Zhuang Autonomous Region People’s Republic of China; 2 Key Laboratory of Early Prevention and Treatment for Regional High Frequency Tumor (Gaungxi Medical University), Ministry of Education, Nanning, 530021 Guangxi Zhuang Autonomous Region People’s Republic of China

**Keywords:** Cervical neoplasm, Deep learning, Segmentation, Meta-analysis, Computed tomography, Radiotherapy

## Abstract

**Background:**

This paper attempts to conduct a systematic review and meta-analysis of deep learning (DLs) models for cervical cancer CT image segmentation.

**Methods:**

Relevant studies were systematically searched in PubMed, Embase, The Cochrane Library, and Web of science. The literature on DLs for cervical cancer CT image segmentation were included, a meta-analysis was performed on the dice similarity coefficient (DSC) of the segmentation results of the included DLs models. We also did subgroup analyses according to the size of the sample, type of segmentation (i.e., two dimensions and three dimensions), and three organs at risk (i.e., bladder, rectum, and femur). This study was registered in PROSPERO prior to initiation (CRD42022307071).

**Results:**

A total of 1893 articles were retrieved and 14 articles were included in the meta-analysis. The pooled effect of DSC score of clinical target volume (CTV), bladder, rectum, femoral head were 0.86(95%CI 0.84 to 0.87), 0.91(95%CI 0.89 to 0.93), 0.83(95%CI 0.79 to 0.88), and 0.92(95%CI 0.91to 0.94), respectively. For the performance of segmented CTV by two dimensions (2D) and three dimensions (3D) model, the DSC score value for 2D model was 0.87 (95%CI 0.85 to 0.90), while the DSC score for 3D model was 0.85 (95%CI 0.82 to 0.87). As for the effect of the capacity of sample on segmentation performance, no matter whether the sample size is divided into two groups: greater than 100 and less than 100, or greater than 150 and less than 150, the results show no difference (*P* > 0.05). Four papers reported the time for segmentation from 15 s to 2 min.

**Conclusion:**

DLs have good accuracy in automatic segmentation of CT images of cervical cancer with a less time consuming and have good prospects for future radiotherapy applications, but still need public high-quality databases and large-scale research verification.

**Supplementary Information:**

The online version contains supplementary material available at 10.1186/s13014-022-02148-6.

## Background

Cervical cancer is the second most common cancer in women aged 15–44 years worldwide, second only to breast cancer in incidence, and the incidence rate and mortality rate are on the rise in recent years [1]. With an annual incidence of about 500,000, more than half of whom die from the disease, cervical cancer is the main reason for the worldwide cancer burden [2]. In many developing countries, most cases of cervical cancer are locally advanced cervical cancer (LACC) when diagnosed [3].

Radiation therapy (RT) was a non-surgical option for lots of varieties of cancer. Likewise, RT is an effective way to improve the survival rate of patients with cervical cancer [4, 5], especially for patients with LACC and those whose physical condition is not suitable for surgery. The preferred approach to radiotherapy for LACC is intensity-modulated radiotherapy (IMRT). To achieve optimal treatment efficacy, for the target area, the radiation dose needs to be increased; and for the surrounding normal tissues and organs, the damage needs to be reduced. Therefore, the key to successful implementation of IMRT is accurate mapping of clinical target volume (CTV) and organs at risk (OARs) [6, 7]. Today, manual segmentation of CTV by a physician is still the standard, but it is a time-consuming and fatiguing task that takes an experienced physician at least 30 min. Even with guidelines, different doctors have different habits, and even the same doctor may have different segmentation results at different times, and there was also literature reported interobserver differences [8]. It is important to note that most CTV does not have clear borders (unlike OAR, which mostly have clear borders) and their contours include not only the apparent lesion volume but also the regional lymph nodes and other suspected pathways of tumor spread [6, 7]. The CTV is largely dependent on individual differences, lesion location, and cancer stage, moreover, even patients with the same stage have different extents of tumor infiltration and lymphatic involvement. All of the above-mentioned will lead to different results of segmentation.

Compared to manual segmentation, automatic segmentation has shown great potential since it was proposed, such as reducing physician burden, decreasing patient waiting time, and improving cancer treatment. During IMRT for Cervical Cancer, the dramatic anatomical changes also require advanced adaptive radiotherapy (ART) strategies [9]. Meanwhile, in low-and middle-income areas and areas with limited medical care, it is difficult to implement radiotherapy according to the guidelines. In this case, the emergence of automatic segmentation can improve the level of local and global medical care [5].

Traditional automatic segmentation methods, such as traditional supervised machine learning and unsupervised machine learning approaches, based on Atlas models and based on statistical models [10], these two methods can obtain great segmentation results, but the segmentation results still require very time-consuming manual editions by the doctor. Unfortunately, both methods have a limitation, in which they cannot handle large differences between different images and different patients [11]. Although a large amount of data can solve this problem, we all know that medical databases, although large in volume, suffer from diversity (i.e., different types, different devices, large differences in data quality, and individual differences of patients), even, many factors can't be dealt with. Therefore we need to use a limited available sample size to achieve the expected results [12, 13]. Thus, all these factors above result in the development of deep learning (DL) networks.

Although DL networks have been around since the 1940s, it was only in 2006 that deep learning emerged as a branch of machine learning, and known as one of the top ten technological breakthroughs since 2013 [12]. Initially, image segmentation by DLs was done with the convolutional neural network (CNN). The CNN usually consists of convolutional layers, pooling layers, and fully connected layers, and its complex structure leads to a large enough sample size and a lot of time, as well as adequate computational capabilities to train the model. Furthermore, CNN has a limitation on image size because of the fixed number of nodes. This problem was then solved by the emergence of the fully convolutional network (FCN), which uses a convolutional layer instead of CNN’s fully-connected layer so that FCN model can handle any image size. In addition, FCN improves segmentation efficiency over CNN because of its skip-connections. Unfortunately, there is also a problem that the multiplier up-sampling in the model is too big, resulting in Insufficient contexts information integration and decrease in segmentation accuracy. The most popular medical image segmentation FCN architecture is the U-net today, which use equal number of up-sampling convolutional layers and down-sampling convolutional layers, and the up-sampling layer can accept the features extracted from the corresponding down-sampling layer because there is a skip-connection in each corresponding layer, so that the segmentation accuracy can be improved. The U-net enables end-to-end training without the need for large numbers of training samples and pre-training [14–17]. With the development of CNN, FCN, and U-net, the accuracy of medical image segmentation has been greatly improved, suggesting that we have entered the development of the fourth generation of segmentation algorithms [13].

Although many reviews have reported the performance of DLs for image segmentation, some previous authors also conducted meta-analyses on the performance of DLs in glioma [18] and head and neck tumor [19] segmentation. However, there is still a lack of comprehensive review and meta-analysis on cervical cancer segmentation. Therefore, this paper aims to investigate the performance of DLs in the segmentation of cervical cancer. Through this paper, highlights the current state and limitations of the field and makes recommendations for research in the future.

## Methods

The systematic review and meta-analysis was conducted in accordance with the Preferred Reporting Items for Systematic Reviews and Meta-Analyses (PRISMA) statement. This study was registered in PROSPERO prior to initiation (CRD42022307071).

### Search strategy

This systematic review and meta-analysis reviews papers on the development or validation of DLs for segmentation of cervical cancer computed tomography (CT) images of CTV or OARs. Publications were retrieved from MEDLINE (accessed via PubMed), The Cochrane Library, Embase, and Web of science, until November 2021. We used ("deep learning", OR "convolutional neural network") AND (Uterine Cervical Neoplasms OR Cervical cancer) as the search strategy, and followed specific search techniques for each database. Language is restricted to English. While there are no publication date restrictions. The full search strategies are available in the Additional file 1.

### Selection criteria and data extraction

Screening of the title, abstract and full text of the paper by two researchers (YC) and (QL), respectively. Discussing and resolving disagreements between the two researchers, the remaining disagreements were resolved jointly with the participation of a third researcher (LJ). All researchers extracted data as follows: (a) publication date and first author; (b) size of training set; (c) whether there was an internal/external validation; (d) CT scan parameters; (e) architecture of the DLs; (f) study design, including segmentation strategy; (g) the DSC score of DLs; (h) time of segmentation. Then, the Cross-validation is performed after finishing extracting data. The inclusion criteria were as follows: (1) develop or validate DLs for segmentation of cervical cancer CT images of CTV and/or OARs; (2) the article reports the structure of DLs, the size of the training set, the size of the validation set, the size of the test set and the DSC score of segmentation; (3) evaluation of segmentation results of DLs by senior oncologists or radiologists. The exclusion criteria were as follows: (1) non-deep learning models; (2) no relevant data reported; (3) animal experiments were excluded; (4) reviews, conference, meta-analysis, and duplicate publication.

### Quality assessment

We evaluated the included articles in this paper with reference to the transparent reporting of a multivariable prediction model for individual prognosis or diagnosis (TRIPOD) [20].

### Statistical analysis

We used Stata software (version 15.1) for meta-analysis. When estimating the overall effect size of the current DLs, for the Meta-analysis, we used a random effects model. As for the normally distributed, data, it is shown as mean with ± SD. By contrast, the abnormally distributed data is displayed as the median with a range (min–max). Statistical tests were considered significant when *p* < 0.05.

The Dice Similarity Coefficient (DSC) [8, 21] was used to evaluate DLs models. The DSC is defined as follows:

$$DSC = \frac{{2\left| {X \cap Y} \right|}}{{\left| {\text{X}} \right| + \left| {\text{Y}} \right|}}$$

where X = {X1, …, Xn} and Y = {Y1, …, Yn} are two finite point sets. X is the predicted mask, and Y is the ground truth. ∣X ∩ Y∣represents the intersection of X and Y and∣X∣ + ∣Y∣represents the union of X and Y.

The Higgins I^2^ test was used to examine the heterogeneity of the included studies. Higgins I^2^ test was able to quantify inconsistency among included studies. When the values > 75% indicated a high degree of heterogeneity between groups. When the values between 25 and 50% indicated moderate heterogeneity between groups. When the value is less than 25% indicated no heterogeneity. We also did subgroup analyses according to the size of the sample, type of segmentation (two dimensions and three dimensions), and three OARs (bladder, rectum, and femur).

The funnel plot and the Egger’s publication bias test were generated with Stata 15.1 to show possible publication bias.

## Results

### Study selection and characteristics

**Fig. 1:**
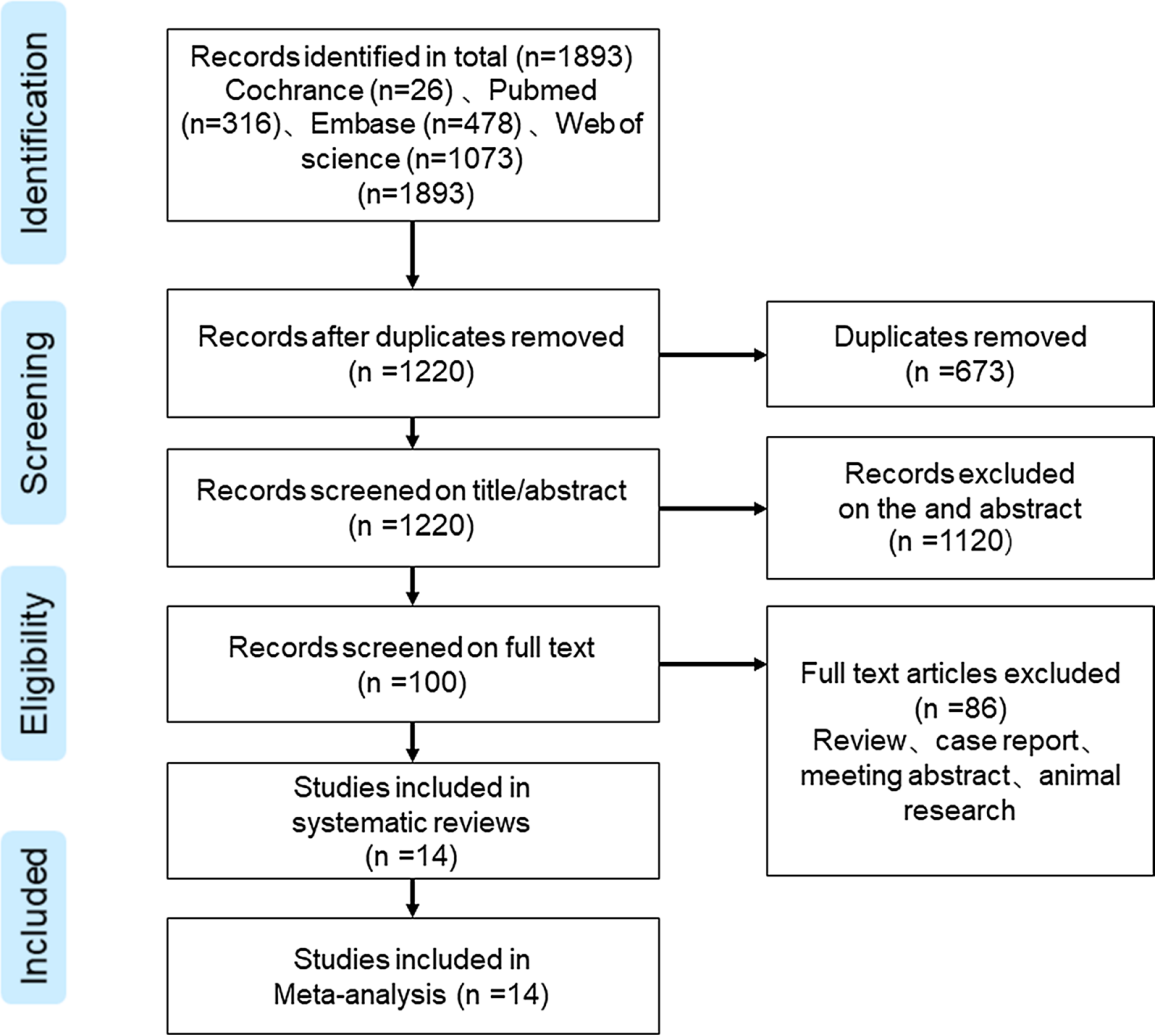
PRISMA flowchart of the eligible studies

A total of 1893 articles were retrieved, of which MEDLINE is 316 (accessed via PubMed), The Cochrane Library is 26, Embase is 478, and Web of science is 1073. After 673 duplicate articles were deleted, 1220 articles remained. Of these, 1120 articles were excluded as irrelevant after viewing the title and abstract. Then, two researchers conducted a full-text review of 100 articles, of which 86 records were excluded and 14 articles were included in the study, according to the inclusion and exclusion criteria (Fig. 1). These 14 articles all focus on segmentations cervical cancer CT images for CTV and/or OARs by DLs. The characteristics of the included article are shown in detail in Table 1.

**Table 1:** The characteristics of the included studies, and structure and outcome of DLs of the included studies

First author	Year (mouth) of publication	Size of training set	Size of test set	Internal/external validation	CT scan parameters
Liu [6]	2020 (October)	210	27	No	Brilliance CT Big Bore (Philips Healthcare, Best, the Netherlands) 512* 512 pixels and 5-mm thickness Input image intensity to—1024 HU and 1024 HU
Wang [4]	2020	100	25	No	5-mm thickness
Shi [7]	2021	308	154	No	SIEMEMS Somatom Definition AS Non-contrast CT scans Software Version of “syngo CT VA48A” 512 × 512 pixels and 5-mm thickness input image intensity to-1000 HU and 600 HU
Rigaud [9]	2021	255 training 61 validation	62 internal test 30 outside test	Yes	Philips Brilliance Big Bore (n = 211), iCT (n = 1), or MX-8000 (n = 4); Siemens Biograph40 (n = 1) or Somatom Definition Edge, (n = 3); GE Light-Speed (n = 160), Discovery, (n = 19), or BrightSpeed (n = 1); Toshiba Aquilion/LB, (n = 2); and unknown (n = 6) 512 × 512 pixels and 3 mm thickness Median (range) peak kilo voltage output of 120 kVp and a median exposure of 300 mAs
Rhee [5]	2020	406	140 internal test 30 outside test	Yes	The CT scans had pixel sizes in the transverse plane that ranged from 0.754 to 1.367 mm and slice thicknesses from 2.0 to 3.0 mm except for 8 CT scans (3 were 5 mm, 3 were 4 mm, 1 was 1.5 mm, and 1 was 1.0 mm thick)
Ju [11]	2021	102 training 11 validation	20	Yes	SIMENS SOMATOM Definition AS 512 × 512 pixels and 5 mm thickness The scanning parameters were 120 kV tube voltage, 400 mAs tube current
Zhang [22]	2020	73	18	No	Brilliance CT Big Bore (Philips Healthcare, Best,the Netherlands) 512 × 512 pixels and 2 mm thickness The average in-plane resolution of the CT slices is 1.11 mm
Sartor [23]	2020	65	10	No	The CT image scanned by pet-CT (Philips Gemini TF, GE Discovery 690, and GE Discovery MI) intra-venous contrast 2.5 mm or 3 mm thickness
Liu [8]	2020 (January)	77 training 14 validation	14	Yes	Philips Brilliance Big Bore 512 × 512 pixels and 5 mm thickness
Liu [24]	2021	237	20	No	Brilliance CT Big Bore (Philips Healthcare, Best, the Netherlands) 512 × 512 pixels
Hu [25]	2021	50 training 10 validation	10	Yes	3 mm slice thickness
Chang [26]	2021	41	10	No	512 × 512 pixels HU -1000 and 2000
Mohammadi [27]	2021	73 training 10 validation	30	Yes	A volumetric HI Speed Dual Slice CT scanner (GE Healthcare, USA) 512 × 512 pixels and 3 mm thickness
Ju [28]	2020	80	20	No	not reported

First author	Year (mouth)of publication	Architecture of the DLs	2D versus 3D	Study design, including segmentation strategy	The DSC score of DLs	Time used for segmentation
Liu [6]	2020 (October)	The DpnUnet that the DPN architecture replaced the whole U-Net encoder part	2.5D	CTV Bladder Bone marrow Left femoral head Right femoral head Rectum Bowel bag Spinal cord	0.86 ± 0.04 0.91 ± 0.05 0.85 ± 0.06 0.90 ± 0.03 0.90 ± 0.02 0.82 ± 0.07 0.85 ± 0.03 0.82 ± 0.05	15 s
Wang [4]	2020	A 3D CNN base on Unet	3D	CTV Bladder Femoral-head-right Femoral-head-left Small intestine Rectum	0.86 ± 0.02 0.91 ± 0.06 0.88 ± 0.05 0.88 ± 0.04 0.86 ± 0.04 0.81 ± 0.04	2 min
Shi [7]	2021	RA-CTVNet consists of three main components: backbone, area-aware reweight strategy and recursive refinement strategy	3D	CTV	0.792 ± 0.073	2 min
Rigaud [9]	2021	2D model DeepLabV3 + with Xception backbone (Google)	2D	OARs	Validation: 0.67–0.96 Internal test sets: 0.71–0.97 External test sets: 0.42–0.92	NR
				CTV	Internal test sets: 0.88 External test sets: 0.81	
		2-step 3D Unet	3D	OARs	Validation: 0.66–0.96 Internal test sets: 0.70–0.97 External test sets: 0.37–0.93	NR
			3D	CTV	Internal test sets: 0.87 External test sets: 0.82	
Rhee [5]	2020	Inception-ResNet-V2(classification)				
		3D V-Net (segmentation)	3D	Primary CTV Bladder Rectum Femur, left Femur, right Kidney, left Kidney, right Pelvic bone Sacrum L4 vertebral body L5 vertebral body	0.86 ± 0.08 0.89 ± 0.09 0.81 ± 0.09 0.94 ± 0.03 0.93 ± 0.04 0.94 ± 0.02 0.95 ± 0.02 0.93 ± 0.02 0.91 ± 0.02 0.91 ± 0.15 0.90 ± 0.15	NR
		2D FCH-8 s (segmentation)	2D	Nodal CTV: PAN CTV: Spinal cord	0.81 ± 0.03 0.76 ± 0.09 0.90 ± 0.02	NR
Ju [11]	2021	Dense V-Net: a deep learning network that integrates two deep learning models of Dense Net and V-Net	3D	CTV	0.82 ± 0.03	NR
Zhang [22]	2020	DSD-UNET (a 3D CNN architecture that is based on the 3D U-Net architecture with incorporation of residual connection, dilated convolution and deep supervision)	3D	CTV Bladder Small intestine Sigmoid Rectum	0.829 ± 0.041 0.869 ± 0.032 0.803 ± 0.058 0.645 ± 0.079 0.821 ± 0.050	20 s
Sartor [23]	2020	A fully-convolutional 3D segmentation network. The output from the network is one channel per organ class with softmax activation plus one channel for the background class	3D	CTVN Femoral Head R Femoral Head L Bladder Bowel bag	0.81 0.92 0.91 0.83 0.86	NR
Liu [8]	2020 (January)	The UNET while the convolutional layers in the U-Net are replaced by Context Aggregation Blocks	2D	Bladder Bone Marrow Femoral Head Left Femoral Head Right Rectum Small Intestine Spinal Cord	0.924 ± 0.046 0.854 ± 0.054 0.906 ± 0.031 0.900 ± 0.023 0.791 ± 0.032 0.833 ± 0.030 0.827 ± 0.063	4.2 s
Liu [24]	2021	DpnUNet (Multicenter Blinded Randomized Controlled Validation)	3D 2D	CTV CTV	0.88 ± 0.03 0.90 ± 0.02	NR
Hu [25]	2021	U-Net	2D	CTV	0.89 ± 0.09	NR
Chang [26]	2021	3D U-Net with bidirectional Convolutional LSTM	3D	CTV Bladder Bowel Foley GTV Rectum Sigmoid Uterus	0.8717 0.8696 0.7227 0.9508 0.718 0.7675 0.7331 0.9326	NR
Mohammadi [27]	2021	ResU-Net (a combination of ResNet and Unet model)	2D	Bladder Rectum Sigmoid	0.957 ± 0.037 0.966 ± 0.015 0.930 ± 0.033	1.5 s
Ju [28]	2020	Dense V-Network algorithm based on a merger of Dense Net and V-Net	3D	Bladder Intestine Rectum Femur-R Femur-L Cord	0.95 0.87 0.87 0.92 0.92 0.87	3 min (Including segmentation of all organs and post-processing of results)

*CTV* Clinical target volume, *OARs* Organ-at-risks, *DLs* Deep learning algorithms, *2.5D* The model was designed as a 2.5D architecture by assigning three adjacent slices into the three channels. The output was the delineation result of the middle slice, *Nodal CTV* Pelvic lymph node CTV, *PAN CTV* Para-aortic lymph node CTV, *CTVN* Clinical target volume of lymph nodes, *GTV* Gross tumor volume, *NR* No report

### Meta‑analysis results

#### CTV

**Fig. 2:**
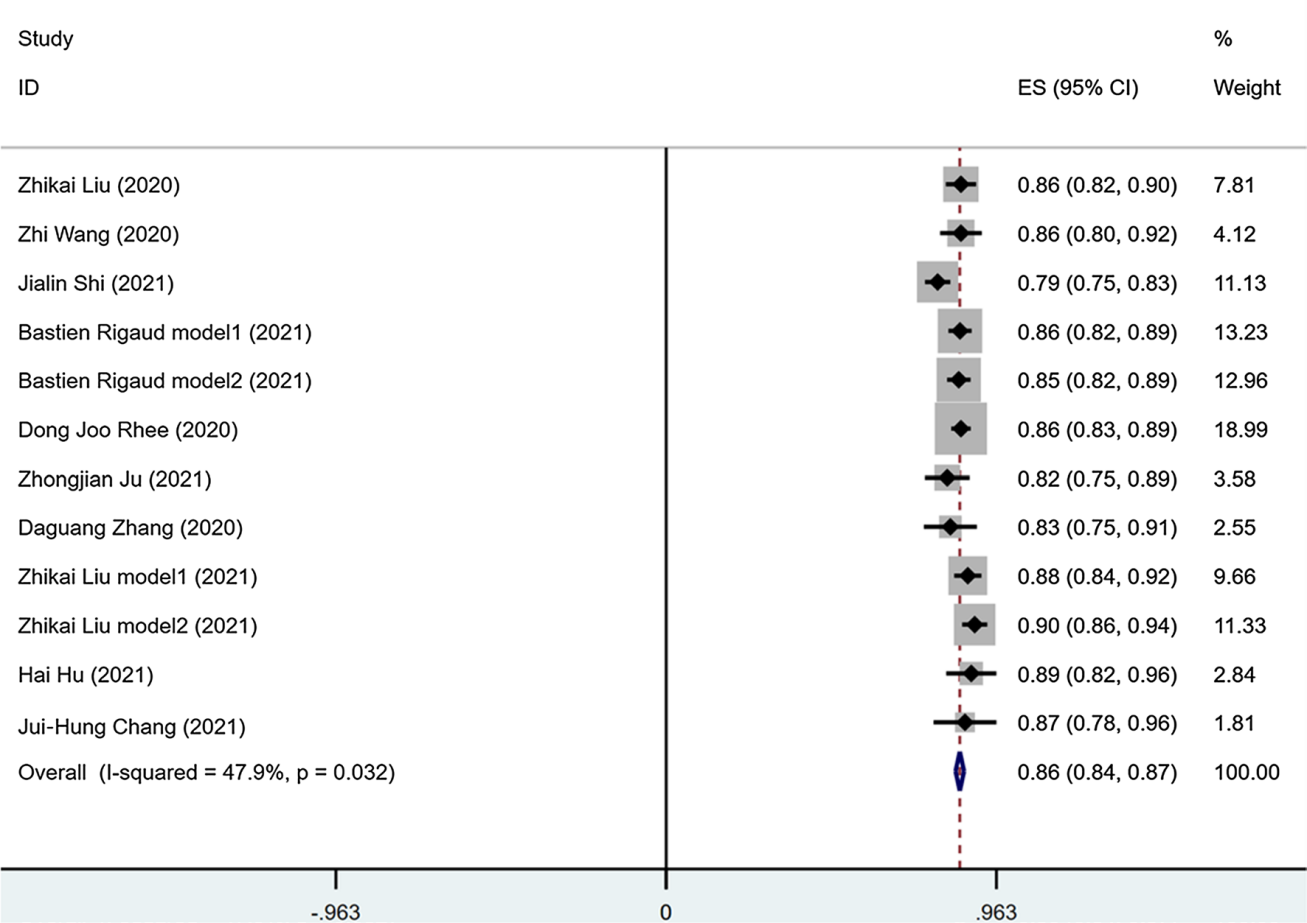
Forest plot of the accuracy of segmentation of cervical cancer. Legend: DSC, dice similarity coefficient; CI, confidence interval. Forest plot shows that the performance of the CTV are centered around a DSC of 0.86 with a 95%CI ranging from 0.84 to 0.87

**Fig. 3:**
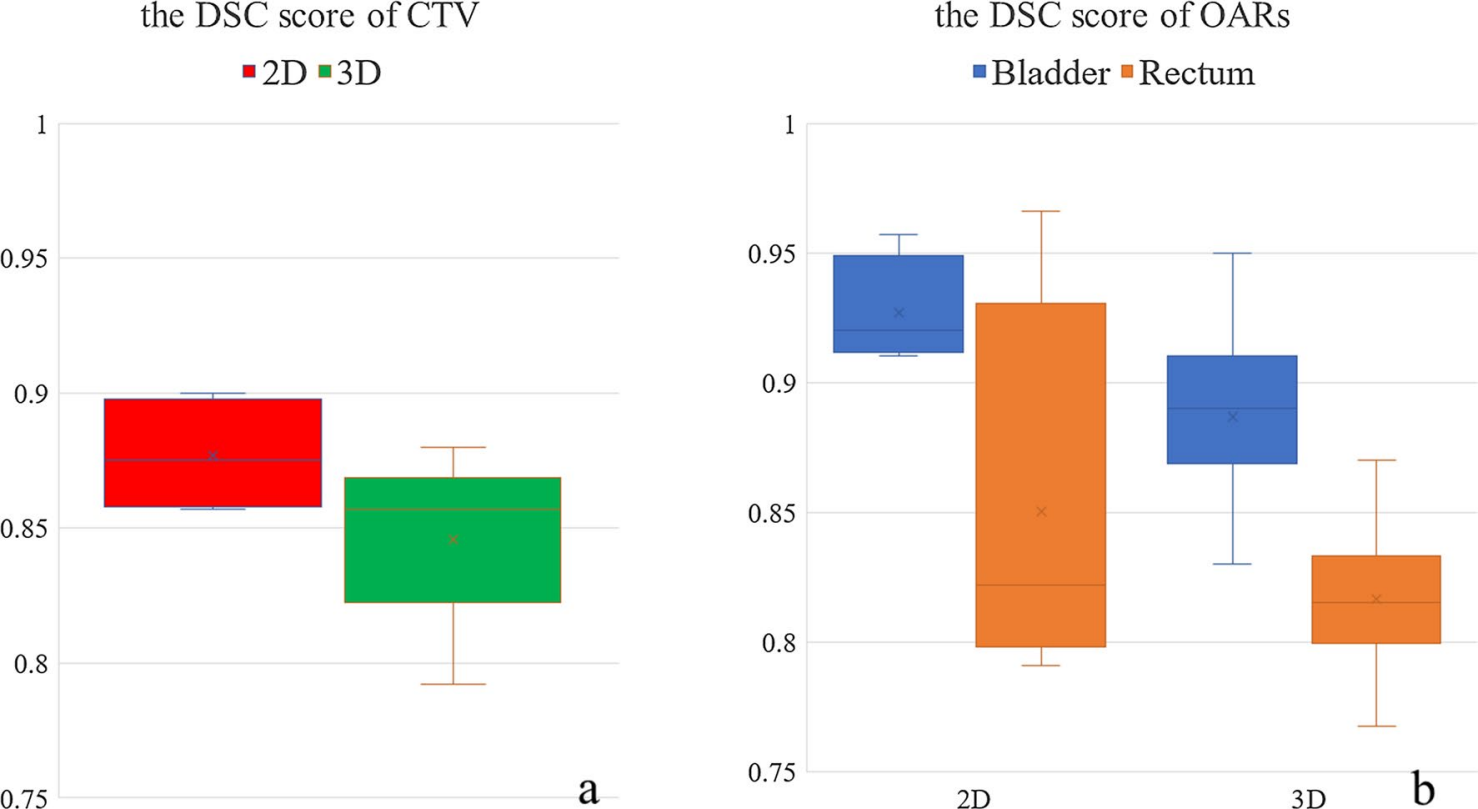
Box plot results of DSC score of CTV and OARs in cervical cancer patients. **a** dice scores of 2D and 3D models of the CTV; **b** dice scores of 2D and 3D models of the OARs. DSC, dice similarity coefficient; CTV, clinical target volume; OARs, organs at risk; 2D, two dimensions; 3D, three dimensions

10 of the 14 studies with a total of 12 DLs segmented the CTV for cervical cancer, among them, 4 papers reported time for segmentation from 15 s to 2 min. The pooled effect of DSC score was 0.86 (95%CI 0.84 to 0.87), Higgins I^2^ is 47.9% that is between 25 and 50%, with moderate heterogeneity (Fig. 2). For the performance of segmented CTV by two dimensions (2D) and three dimensions (3D) model, the DSC score value for 2D model was 0.87 (95%CI 0.85 to 0.90), while the DSC score for 3D model was 0.85 (95%CI 0.82 to 0.87). There was significant difference between the two groups (*p* = 0.039) (Fig. 3a). To investigate the effect of the capacity of sample on segmentation performance, no matter whether the sample size is divided into two groups: greater than 100 and less than 100, or greater than 150 and less than 150, the results show no difference (*P* > 0.05).

#### OARs

For OARs, included articles had focused on the bladder, rectum, femur, L4 vertebral body, L5 vertebral body, sigmoid colon, etc. Due to the number of studies that reported OARs, this article focuses on only three OARs: bladder, rectum, and femur. Two of the included articles reported that splitting the OAR takes 1.5 s and 4.2 s.

#### Bladder

For the bladder, there were 10 articles in the included studies, of which 11 models mentioned segmentation of the bladder, the total effect of DSC score was 0.91 (95%CI 0.89 to 0.93). Higgins is I^2^ = 48.5% with moderate heterogeneity. For the 2D model and 3D model of the segmented bladder, the pooled effect of DSC score of 2D model and 3D model were 0.93 (95%CI 0.91 to 0.96), and 0.90 (95%CI 0.87 to 0.92) respectively, with a significant difference between the two groups (*p* = 0.018) (Fig. 3b).

#### Rectum

For the rectum, there were 9 articles included, of which 10 models mentioned segmentation of the rectum, the pooled effect of DSC score was 0.83 (95%CI 0.79 to 0.88). Higgins I^2^ is 86.0% with a high degree of heterogeneity. For the 2D model and 3D model of the segmented rectum, the total effect of DSC score of 2D model and 3D model were 0.85 (95%CI 0.77 to 0.94) and 0.82 (95%CI 0.80 to 0.84), with a significant difference between the two groups (*p* = 0.000) (Fig. 3b).

#### Femoral head

For the femoral head, there were 7 articles in the included studies, of which 8 models mentioned segmentation of the femoral head, the pooled effect of DSC score of the femoral head was 0.92 (95%CI 0.91 to 0.94). Higgins I^2^ is 28.0% with moderate heterogeneity,

We also compare the performance of DLs segmentation of different OARs and the results show: (1) no difference in bilateral femoral head (*P* > 0.05); (2) performance of DLs for segmentation of the femoral head is superior to that for segmentation of the bladder and rectum (*P* < 0.05); (3) performance of DLs for segmentation of the bladder is superior to that for segmentation of the rectum (*P* < 0.05).

### Risk of bias

**Fig. 4:**
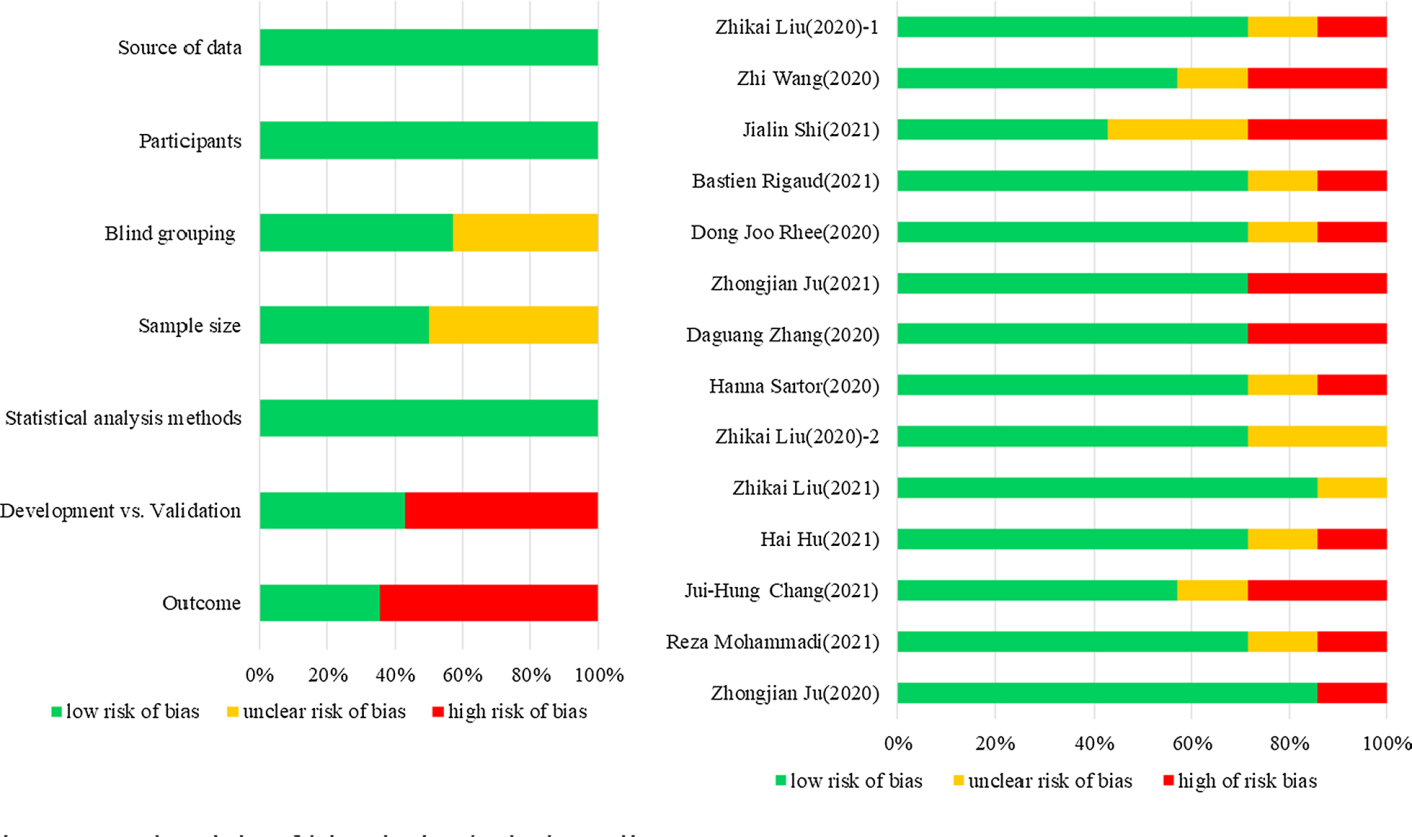
The risk of bias in included studies

The risk of bias was assessed in 14 included studies according to the TRIPOD tool. Results showed 12 studies were rated as high risk (Fig. 4), the main reasons are the following two: (1) validation results for models are not reported or validation groups are not set; (2) reported applicability of DLs segmentation results for clinical.

### Publication bias

**Fig. 5:**
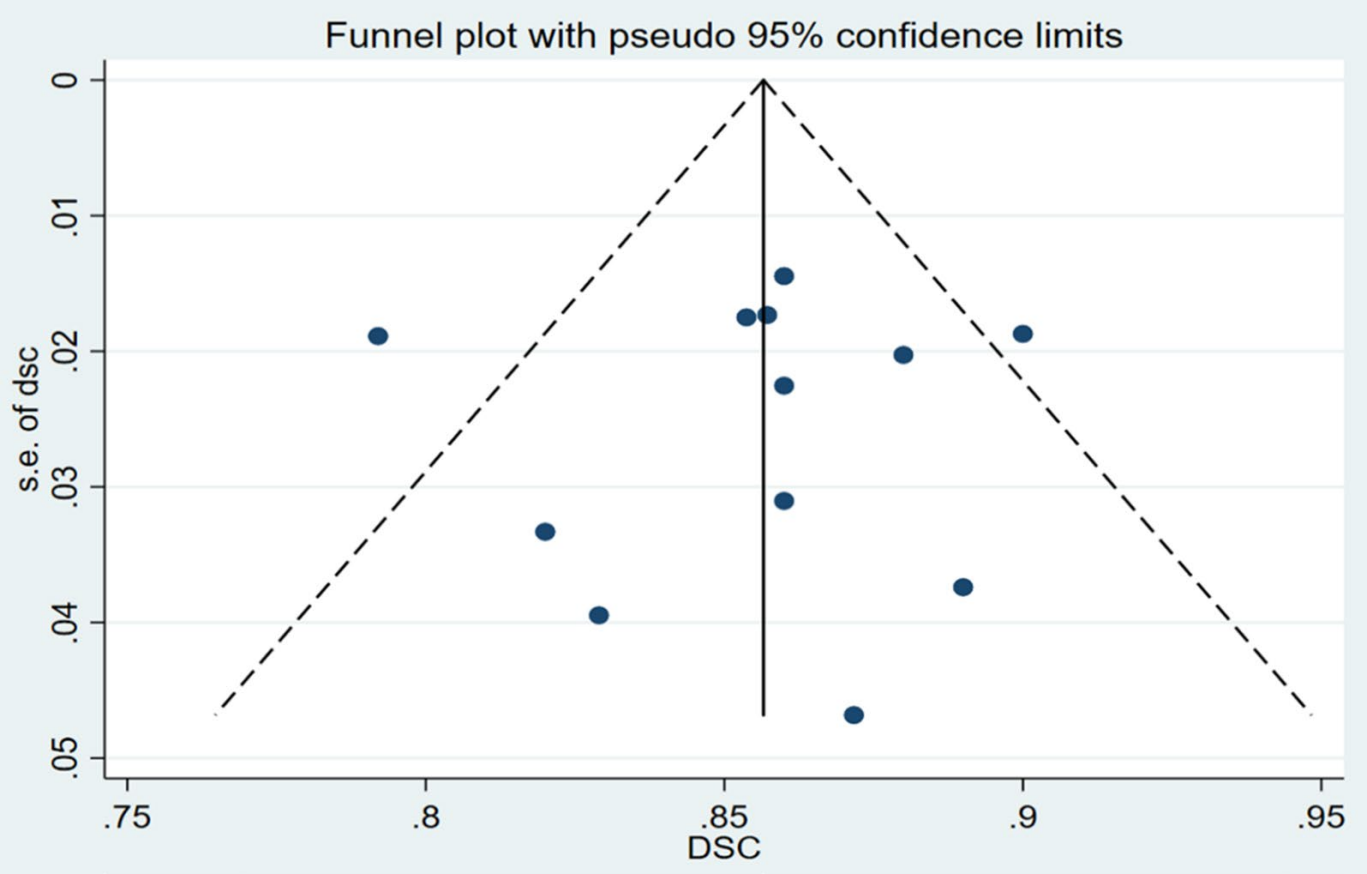
Funnel plot of the included studies

**Fig. 6:**
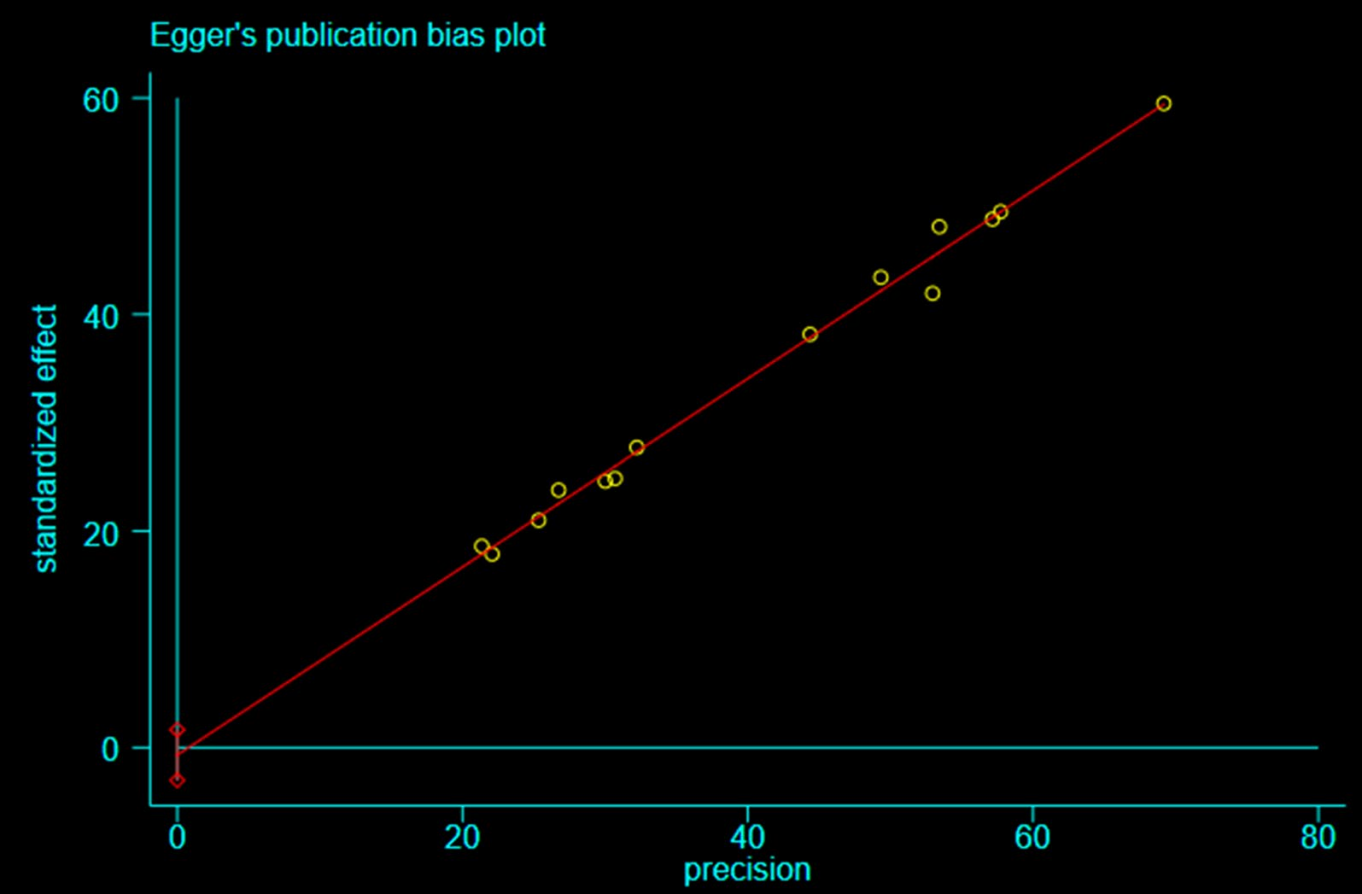
Egger’s publication bias plot

The studies included in the funnel plot were the 12 studies that reported CTV, the funnel plot had a symmetrically distributed shape and the Egger’s publication bias test show the *P* = 0.531 > 0.05, implying no publication bias in the included studies (Figs. 5, 6). For details of the publication bias of included studies that reported OARs are available in the Additional file 1.

## Discussion

This paper systematically reviews various DLs models for the segmentation of cervical cancer CTV and OARs. in which the models are almost all U-net and its variants, and despite some heterogeneity, they still show excellent performance of DLs with no significant publication bias.

At present, manually delineating CTV is still a very time-consuming and discrepancy-prone task. For a physician, it often takes half an hour to delineate the CTV, while for DLs it takes only 15 s to 2 min to complete [4, 6, 7, 22]. The results of the evaluation of the metrics show that the current performance of deep learning segmentation of cervical cancer CTV and OARs can achieve good results (DSC > 0.8), and oncologists also say that the results of segmentation by DLs can be used directly or with minor modifications in clinical RT [5, 6, 8, 23, 24]. Therefore, with computer assistance, it will provide great convenience and optimize the work of clinical radiotherapy.

The results for both CTV and OARs segmentation show that the 2D model has a better performance than the 3D model. As you can imagine, this is an obvious problem for a few main reasons: (a) 3D models have more data to process and require more computational capabilities and more optimized algorithms to handle segmentation issues [26, 29, 30]; (b) Comparing to 3D models, 2D metrics typically provide less bias and do not account for under- contour or over-contouring in the 2D slice [6]; (c) 3D models demand a larger sample size and correctly encode the essential features in the image, and a large number of training samples complicates the computational process of the model and may increase the risk of overfitting [27, 31]. But the lack of training data makes the feature capture process of 3D models more difficult [28]. Thus, 2D models can show superior performance to 3D at this stage.

It is worth noting that 2D model has its disadvantages: the loss of data between slices, while 3D models use the entire volume of the image rather than individual slices. Oscar noted that in prostate cancer detection and segmentation tasks, 3D models tend to show better performance than 2D [32]. On the one hand, 2D models have started to appear saturated in many areas, on the other hand, 3D models have many advantages that 2D models do not have: (a) for anatomical structures and lesions with wide variations or irregular shapes, 3D models can show superior performance compared to 2D models if more kinds of data can be collected [6, 31]; (b) compared with 2D-based RT plans, 3D-based plans can reduce the dose to patients and improve their prognosis [22, 33–36]. These two advantages also show that 3D is the need for the advancement of medical images, and it is also the necessity of developing 3D models. In addition, it is noted that we are currently evaluating the segmentation results of the 3D model in cross-section with a 2D parameter DSC, because the oncologists are also evaluating it. In this way, which will underestimate the performance of the 3D model. But under such conditions, the 3D DL model can also reach a satisfactory segmentation result, and the performance of the 3D model in the future is worthy of our expectation.

For OARs, we are more concerned about the organs adjacent to the cervix, like the bladder and rectum, because of their anatomical location, they are prone to receive radiation and cause radiological sequelae, such as bladder fistula, cystitis, proctitis, etc. All of the above complications will affect the patient's quality of life afterward, and accurate delineation of OARs will reduce the probability of postoperative complications in patients [37–40]. In this article, DLs segmented both bladder and rectum with good results. The results indicated that segmentation of the bladder performed better than segmentation of the rectum. and the main reasons may be as follows: one is that the bladder contents are urine and the density is more homogeneous, and the other is that the anatomy of the bladder is more rounded and regular, the bladder has comparatively well-defined borders and the bladder has higher contrast with the surrounding tissue [16], so there are enough features for DLs learning and recognition to reach a good DSC. Dazhou Guo proposed to classify OARs into three difficulties by the contrast between OAR and surrounding, and also demonstrated that organs with high contrast with surrounding tissues could achieve higher DSC [41]. In contrast, the borders of the intestine are less clear than those of the bladder, and the main surrounding structures are fatty tissue, which made the intestine lacks contrast with the surrounding tissue [23]. The intestinal contents are not consistent (including air and fecal stones), which also affects the contour of the intestine and its internal density and increase the difficulty of segmentation [5], therefore, the effect of DL in dividing intestines is slightly worse and has a certain difference. Likewise, failure to delineate the femur may lead to complications such as ischemic necrosis of the femoral head and bone marrow suppression after radiotherapy. For segmenting the femoral head excellent results can be achieved, which is superior to the two soft tissues, bladder and rectum. We also observed that the Pelvic bone, L4 and L5 vertebral bodies, which are also bony structures, can also reach DSC 0.9 or higher. The main reason is the great contrast between the bone structure and the surrounding tissue, which is more conducive for DLs to learn and segment [23].

We found no significant effect of sample size on the segmentation results by comparison, suggesting that DLs (U-net) can achieve excellent segmentation performance using small sample training. This may be due to the technique of data augmentation (panning, flipping, deforming, rotating or using DL generation, etc.), or the advantages of the U-net algorithm, although this fact was not statistically verified in this paper [42, 43]. It still shows that U-net can achieve excellent segmentation results with DL models mapped out by training with small samples.

Furthermore, we have to focus on the robustness of U-net, once, deep learning was considered by clinicians to be a "black box algorithm" because of the uncertainty of its results [44]. Nowadays, the same excellent results (DSC > 0.8) have been achieved in several internal and external tests and even in multi-center blinded randomized controlled tests [5, 9, 24]. Moreover, we also noticed that the U-net achieved good segmentation results in glioma [18], head and neck tumors [19], prostate cancer [45], and breast cancer [46]. Despite the differences in segmentation results, as long as they are within the acceptable range of guidelines, it makes sense to have several more treatment options for physicians to choose from and to reduce patient waiting time on the other [47].

However, this paper also has certain limitations: (1) this study only focuses on the segmentation performance of CT images and does not elaborate on MR-based and PET-based, while we know that MRI T2-WI and DWI have very good lesion display power and contouring ability. Therefore, the performance of DLs for segmentation in MR images and PET images needs to be further investigated. (2) This study focuses on only one evaluation metric of DSC, while for segmentation, we have many more metrics to evaluate, such as the Hausdorff Distance (HD), Jaccard Distance (JD), the Deviation of Volume (ΔV), and Sensitivity Index (SI) [22], different metrics have different meanings, and we need to use more metrics to measure the performance of different models in future work. (3) There is a lack of publicly available quality data sets for the task of cervical cancer segmentation. (4) The existing training sets are almost all in the order of a hundred, and it is worthwhile to verify whether there are better results if they exceed the order of a thousand in future larger-scale or multi-center studies.

Nevertheless, we were able to see the very powerful potential of deep learning for cervical cancer image segmentation. Hassanzadeh [48] proposes to use enough 2D data to do 3D segmentation instead of the original 3D data, also achieving higher accuracy and saving at least 75% of time and computation. Linyan Gu proposed the fusion of 2d to 3d (2d UNet +  + ASPP to 3D ResUNet) two-step model, this model not only utilizes the information between levels, but also reduces the rate of missed detections compared to a pure 2D model. It also reduces the training time, compared to a pure 3D model [49]. All of these experiences and methods are worth learning from in the future.

## Conclusions

This systematic review and meta-analysis show that DLs have good accuracy in automatic segmentation of CT images of cervical cancer with a less time consuming and have good prospects for future radiotherapy applications, but still need public high-quality databases, and large-scale research verification.

## Supplementary Information

**Additional file 1. **The search strategy and the additional figure.

## Data Availability

Not applicable.

## Abbreviations

CCRT: Concurrent chemoradiotherapy
FIGO: The International Federation of Gynecology and Obstetrics
CT: Computed tomography
LACC: Locally advanced cervical cancer
RT: Radiation therapy
IMRT: Intensity-modulated radiotherapy
CTV: Clinical target volume
OARs: Organs at risk
ART: Advanced adaptive radiotherapy
CNN: Convolutional neural network
DL: Deep learning
FCN: Fully convolutional network
DSC: Dice similarity coefficient
2D: Two dimensions
3D: Three dimensions
PRISMA: The preferred reporting items for systematic reviews and meta-analyses
RECIST: Response evaluation criteria in solid tumors
