# Supplementary material for: Deep learning in CT image segmentation of cervical cancer: a systematic review and meta-analysis
Source: Radiat Oncol. 2022 Nov 7;17:175. doi: 10.1186/s13014-022-02148-6 (PMC9641941; doi:10.1186/s13014-022-02148-6)
Supplement: Supplementary file 1 — Additional file 1. The search strategy and the additional figure. [file 13014_2022_2148_MOESM1_ESM.docx]

# Search strategy:

## The Cochrance library

#1 (artificial intelligence):ti OR (AI):ti OR (neural network*):ti

#2 (machine learning):ti OR (ensemble):ti,ab,kw

#3 (deep):ti,ab,kw

#4 (deep learning OR deep-learning OR reinforcement learning OR reinforcement-learning OR deep

neural network* OR deep belief network* OR convolutional neural network* OR recurrent neural

network* OR feedforward neural network* OR Boltzmann machine* OR long short-term memory OR

gated recurrent unit OR rectified linear unit OR autoencoder OR backpropagation OR multilayer

perceptron OR convnet OR convolutional learning):ti,ab,kw

#5: #2 AND #3

#6 #1 OR #4 OR #5

#7 cervical cancer

#8 cervix cancer

#9 Uterine Cervical Neoplasms

#10 Uterine Cervical tumor

#11 #7 OR #8 OR #9 OR #10

#12 #6 AND #11

result ：SRs：0 protocol：0 Trials：26

## Pubmed

#1: (((((((((Deep Learning[MeSH Terms) OR (Learning, Deep[Title/Abstract])) OR (Hierarchical Learning[Title/Abstract])) OR (Learning, Hierarchical[Title/Abstract])) OR (artificial intelligence[Title/Abstract])) OR (AI[Title/Abstract])) OR (neural network*[Title/Abstract])) OR (deep belief network*[Title/Abstract])) OR (recurrent neural network*[Title/Abstract])) OR (feedforward neural network*[Title/Abstract])

#2: (((((((((((((((((((((((((((Uterine Cervical Neoplasms[MeSH Terms]) OR (Cervical Neoplasm, Uterine[Title/Abstract])) OR (Cervical Neoplasms, Uterine[Title/Abstract])) OR (Neoplasm, Uterine Cervical[Title/Abstract])) OR (Neoplasms, Uterine Cervical[Title/Abstract])) OR (Uterine Cervical Neoplasm[Title/Abstract])) OR (Neoplasms, Cervical[Title/Abstract])) OR (Cervical Neoplasms[Title/Abstract])) OR (Cervical Neoplasm[Title/Abstract])) OR (Neoplasm, Cervical[Title/Abstract])) OR (Neoplasms, Cervix[Title/Abstract])) OR (Cervix Neoplasms[Title/Abstract])) OR (Cervix Neoplasm[Title/Abstract])) OR (Neoplasm, Cervix[Title/Abstract])) OR (Cancer of the Uterine Cervix[Title/Abstract])) OR (Cancer of the Cervix[Title/Abstract])) OR (Cervical Cancer[Title/Abstract])) OR (Uterine Cervical Cancer[Title/Abstract])) OR (Cancer, Uterine Cervical[Title/Abstract])) OR (Cancers, Uterine Cervical[Title/Abstract])) OR (Cervical Cancer, Uterine[Title/Abstract])) OR (Cervical Cancers, Uterine[Title/Abstract])) OR (Uterine Cervical Cancers[Title/Abstract])) OR (Cancer of Cervix[Title/Abstract])) OR (Cervix Cancer[Title/Abstract])) OR (Cancer, Cervix[Title/Abstract])) OR (Cancers, Cervix[Title/Abstract]))

#3 #2 AND #1

result 316

## Embase

#1 'deep learning'/exp OR 'learning, deep':ab,ti OR 'hierarchical learning':ab,ti OR 'learning, hierarchical':ab,ti OR 'artificial intelligence':ab,ti OR ai:ab,ti OR 'neural network*':ab,ti OR 'deep belief network':ab,ti OR 'recurrent neural network':ab,ti OR 'feed forward neural network':ab,ti

#2 'uterine cervix tumor'/exp OR 'uterine cervical neoplasms':ab,ti OR 'cervical neoplasm, uterine':ab,ti OR 'cervical neoplasms, uterine':ab,ti OR 'neoplasm, uterine cervical':ab,ti OR 'neoplasms, uterine cervical':ab,ti OR 'uterine cervical neoplasm':ab,ti OR 'neoplasms, cervical':ab,ti OR 'cervical neoplasms':ab,ti OR 'cervical cancer':ab,ti OR 'neoplasms, cervix':ab,ti OR cancer,cervix:ab,ti OR 'cervix neoplasms':ab,ti OR 'cervix cancer':ab,ti OR 'cancer of the cervix':ab,ti OR 'cancer of the uterine cervix':ab,ti OR 'uterine cervical cancer':ab,ti OR 'cancer, uterine cervical':ab,ti OR 'cancers, uterine cervical':ab,ti OR 'cervical cancer, uterine':ab,ti OR 'cancer of cervix':ab,ti

#1 AND #2

result 478

## Web of science

#1 TS=(“cervical cancer” OR “Cancer of Cervix” OR “Cancer of the Cervix” OR Cervical Neoplasia* OR Cervix Neoplasm* OR “Neoplastic Cervix” OR ((Cervix OR Cervical) AND (Cancer OR Cancers OR Cancerous OR Carcinoma OR Neoplasia* OR Neoplasm* OR Neoplastic)))

#2 ((((((TS=(deep learning)) OR TS=(hierarchical learning)) OR TS=(artificial intelligence)) OR TS=(neural network*)) OR TS=(deep belief network)) OR TS=(recurrent neural network)) OR TS=(AI)

#1 AND #2

result 1073


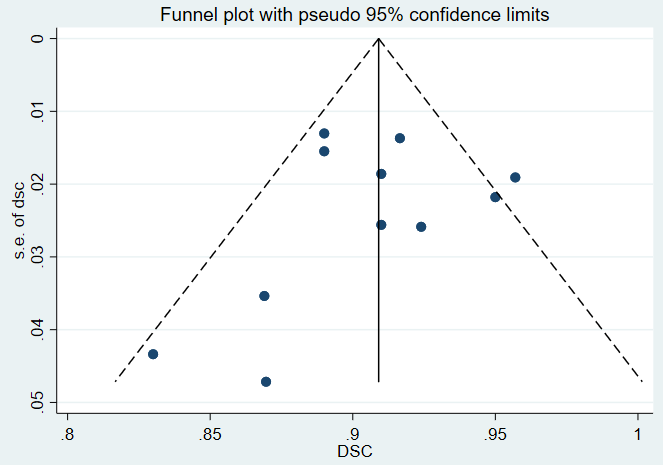


Figure 1. Funnel plot of the included studies that reported segmented bladders.


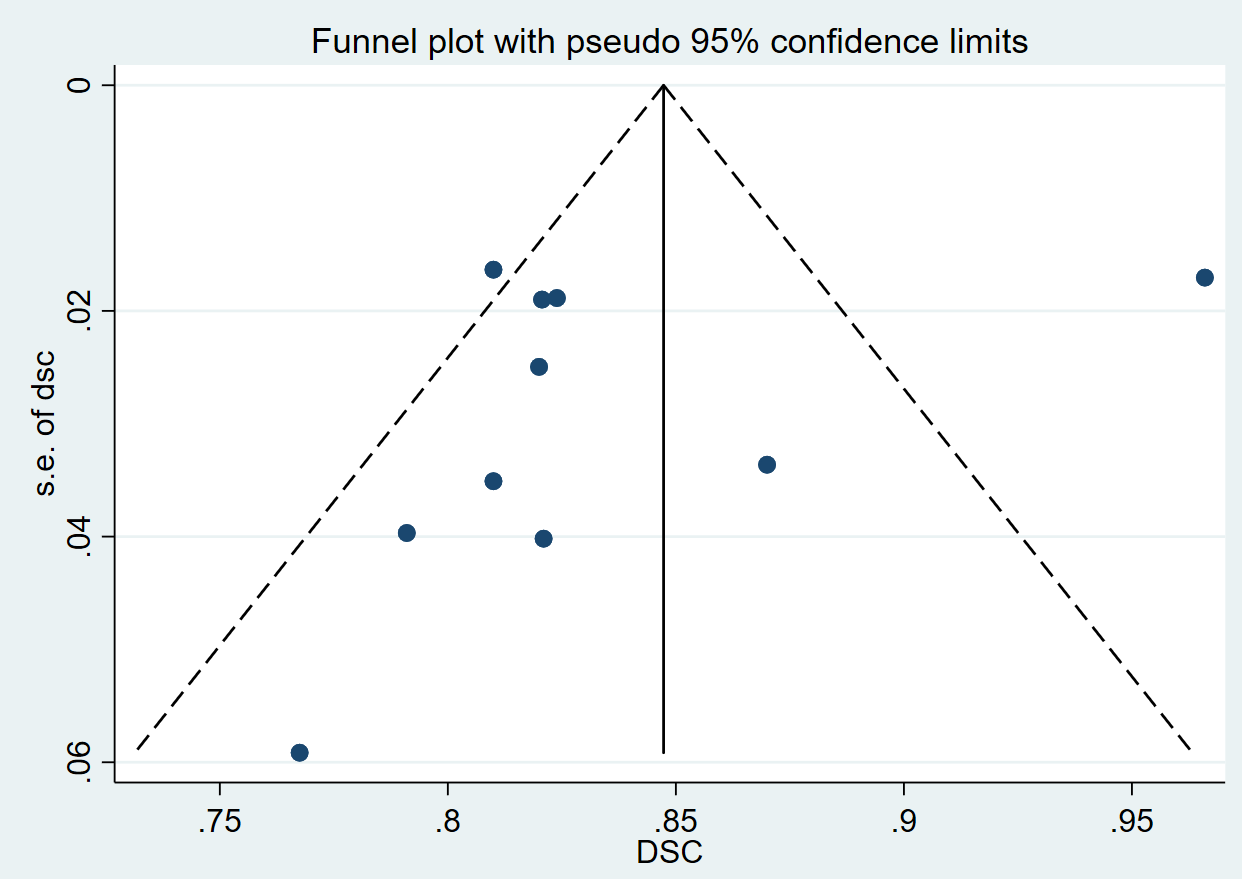


Figure 2. Funnel plot of the included studies that reported segmented rectum.


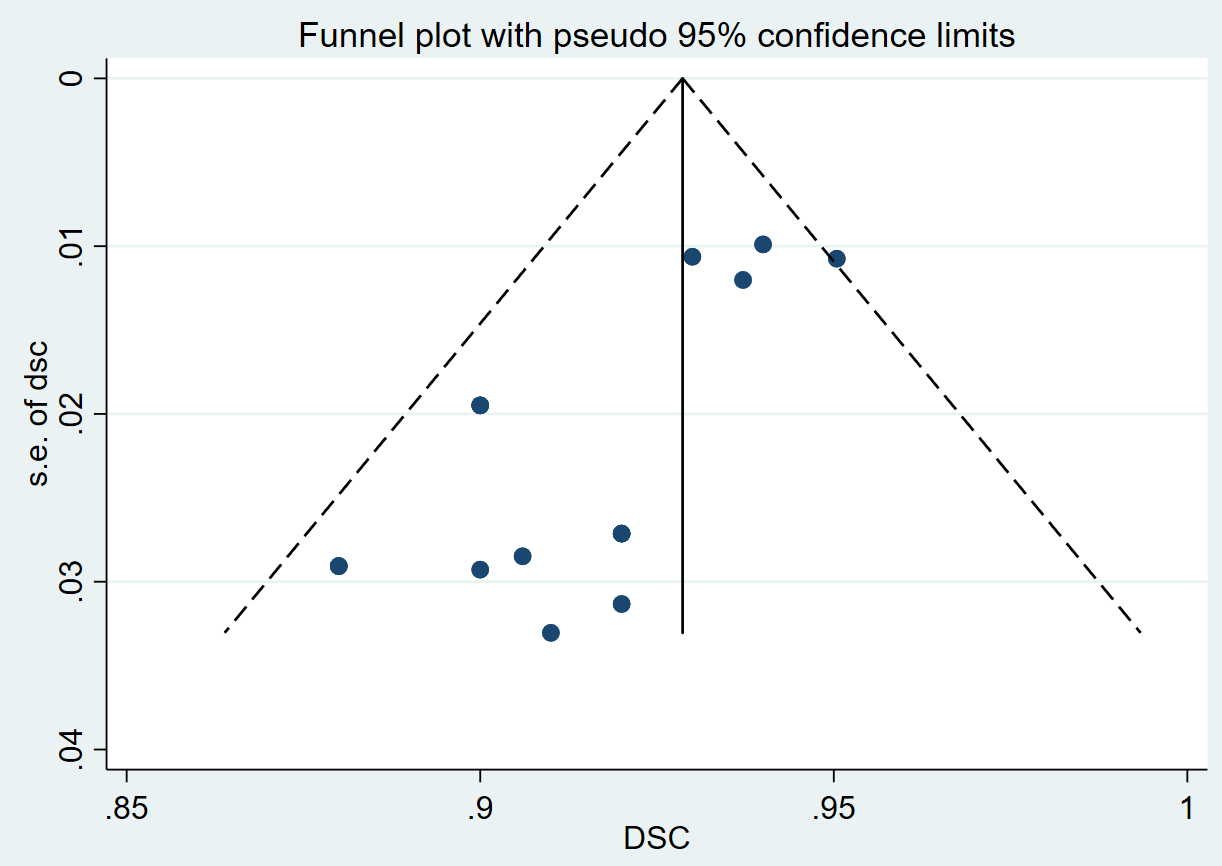


Figure 3. Funnel plot of the included studies that reported segmented femoral head.
